# Proteomics reveals differential adsorption of angiogenic platelet lysate proteins on calcium phosphate bone substitute materials

**DOI:** 10.1093/rb/rbac044

**Published:** 2022-07-05

**Authors:** Richard da Costa Marques, Johanna Simon, Cyril d’Arros, Katharina Landfester, Kerstin Jurk, Volker Mailänder

**Affiliations:** Dermatology Clinic, University Medical Center of the Johannes Gutenberg-University Mainz, 55131 Mainz, Germany; Max Planck Institute for Polymer Research, 55128 Mainz, Germany; Dermatology Clinic, University Medical Center of the Johannes Gutenberg-University Mainz, 55131 Mainz, Germany; Max Planck Institute for Polymer Research, 55128 Mainz, Germany; INSERM, UMR 1229, Regenerative Medicine and Skeleton, ONIRIS, Université de Nantes, 44042 Nantes, France; Biomatlante—Advanced Medical Solutions Group Plc, 44360 Vigneux-de-Bretagne, France; Max Planck Institute for Polymer Research, 55128 Mainz, Germany; Center for Thrombosis and Hemostasis (CTH), University Medical Center of the Johannes Gutenberg-University Mainz, 55131 Mainz, Germany; Dermatology Clinic, University Medical Center of the Johannes Gutenberg-University Mainz, 55131 Mainz, Germany; Max Planck Institute for Polymer Research, 55128 Mainz, Germany

**Keywords:** protein adsorption, calcium phosphates, proteomics, angiogenesis, platelet lysate

## Abstract

Protein adsorption on biomaterials for bone substitution, such as calcium phosphates (CaP), evokes biological responses and shapes the interactions of biomaterials with the surrounding biological environment. Proteins adsorb when CaP materials are combined with growth factor-rich hemoderivatives prior to implantation to achieve enhanced angiogenesis and stimulate new bone formation. However, the identification of the adsorbed proteins and their angiogenic effect on bone homeostasis remain incompletely investigated. In this study, we analyzed the adsorbed complex protein composition on CaP surfaces when using the hemoderivatives plasma, platelet lysate in plasma (PL), and washed platelet lysate proteins (wPL). We detected highly abundant, non-regenerative proteins and anti-angiogenic proteins adsorbed on CaP surfaces after incubation with PL and wPL by liquid chromatography and mass spectrometry (LC–MS) proteomics. Additionally, we measured a decreased amount of adsorbed pro-angiogenic growth factors. Tube formation assays with human umbilical endothelial cells demonstrated that the CaP surfaces only stimulate an angiogenic response when kept in the hemoderivative medium but not after washing with PBS. Our results highlight the necessity to correlate biomaterial surfaces with complex adsorbed protein compositions to tailor the biomaterial surface toward an enrichment of pro-angiogenic factors.

## Graphical Abstract

**Figure:**
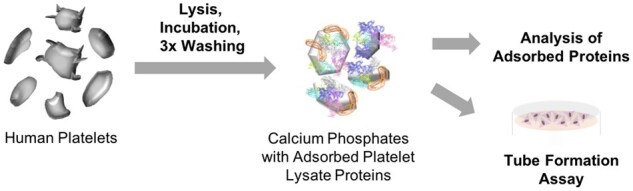

## Introduction

Differences in adsorption characteristics of proteins on biomaterial surfaces must be considered for the engineering of biomaterials to enhance their regenerative potential [1–3]. For bone substitution, biomaterial engineering has put forth a variety of synthetic bone substitute materials [4, 5]. Among these, calcium phosphates (CaP) and other synthetic bone substitute materials are more frequently used for smaller bone defects [5–7]. CaPs represent an absorbable and bioactive biomaterial platform with versatile and promising functionalization approaches to improve regenerative effects after application [8–11]. However, the application of CaPs in larger bone defects has been limited because of their inherent lack of growth factors [6, 12]. This lack of growth factors results in the inefficiency of CaPs to stimulate the formation of new bone [12]. To improve the formation of new bones, a crucial process is an efficient angiogenesis, the sprouting of new blood vessels [12, 13].

Previous studies have suggested the use of hemoderivative materials as growth factor-rich sources. Here, plasma, serum, platelet-rich plasma (PRP), or platelet lysates (PL) are currently combined with synthetic bone substitute materials [14, 15]. These hemoderivative materials are cheap and easily obtainable sources of growth factors compared with single and isolated growth factors [6]. However, combining the promising hemoderivative growth factor sources with bone substitute materials remains a disputable method with controversial results [6, 14, 15].

To understand these controversial results, we critically considered the complexity of proteins in hemoderivatives and the surface-adsorbed proteins that can induce but also inhibit angiogenesis. Activated platelets are well-known modulators of angiogenesis by the differential release of pro- and anti-angiogenic factors from platelet α-granules in an agonist-dependent manner [16–18]. Therefore, resting platelets are an excellent source of a variety of enriched angiogenic factors [19]. Highly abundant and potent pro-angiogenic growth factors stored in platelet α-granules include vascular endothelial growth factor (VEGF), fibroblast growth factor 2 (FGF-2) and platelet derived growth factor AB (PDGF-AB), which are essentially involved in proliferation and migration of endothelial cells in early and late stages of vessel branching, respectively [20]. The chemokine platelet factor 4 (PF4, CXCL4), the matricellular response to injury protein thrombospondin-1 (TSP-1) and endostatin, a C-terminal fragment of collagen type XVIII, represent prominent platelet-derived anti-angiogenic factors, which commonly inhibit the ligation of distinct growth factors, e.g. VEGF, FGF-2, to endothelial cells [21–23]. Presently, the protein adsorption on ‘modified’ bone substitute materials and the biological effect remain poorly studied for combining hemoderivatives with bone substitute materials. Therefore, we revealed the protein adsorption and its impact on angiogenesis in the case of CaP surfaces combined with hemoderivatives.

Here, we present for the first time a label-free liquid chromatography-mass spectrometry (LC–MS) proteomics approach to analyze the complex composition of the adsorbed proteins on CaP surfaces when incubated in the commonly used hemoderivatives citrate plasma (cP), platelet lysate with plasma (PL), and washed PL without plasma components (wPL). Additionally, we determined the concentrations of selected growth factors on the CaP surfaces by enzyme-linked immunosorbent assay (ELISA). Finally, we analyzed the angiogenic potential of preincubated CaP surfaces in tube-formation assays with human umbilical endothelial cells (HUVECs). We report our findings of highly abundant, non-regenerative proteins and single anti-angiogenic proteins on CaP surfaces when utilizing PL and wPL as a hemoderivative source. The adsorbed proteins only led to a non-angiogenic response when the CaP granules were washed before application. Further, we demonstrate that the CaP were only able to stimulate an angiogenic response when kept in the hemoderivative medium.

## Materials and methods

### Human citrate plasma

Human citrate plasma (cP) was provided by the Department of Transfusion Medicine Mainz from healthy donors in accordance with the Declaration of Helsinki. Citrate plasma was generated by plasmapheresis. A plasma pool from ten healthy donors was created, aliquoted and stored at −80°C.

### Freeze–thaw preparation for generating human PL

PL was obtained from human apheresis platelet concentrate by performing a previously published, standardized freeze-thaw lysis method [24]. The human platelet apheresis from ten healthy donors was obtained from the Transfusion Center of the University Medical Center Mainz in accordance with the Declaration of Helsinki. The platelet apheresis from all donors was pooled and aliquoted. The lysis was performed in five freeze–thaw cycles by freezing for 10–40 min at −80°C and thawing for 7.5 min at 37°C. To remove cellular debris, PL was centrifuged at 1410 *g* for 40 min at 22°C (5702R, Eppendorf, Germany). The supernatant was collected, aliquoted and stored at −80°C.

### Platelet preparation for generating human washed PL

Human whole blood was obtained from healthy volunteers in accordance with the Declaration of Helsinki. The study was approved by the Ethics Committee of the University Medical Center Mainz (Study No. 837.302.12; 2018-13290_1). The volunteers did not take any medication for at least 10 days. All donors gave their informed consent before participating in the study. Venous blood was collected and anticoagulated with 10.6 mM trisodium citrate. Platelet isolation and washing were performed as previously published [25]. To bind remaining free calcium, a 0.5 M EGTA solution was added for a final concentration of 2 mM. The blood samples were centrifuged at 200 *g*, for 10 min at room temperature (RT; Allegra X-30R, Beckman Coulter, USA). The supernatant, containing PRP was diluted with the same volume of CGS buffer (120 mM NaCl, 12.9 mM Tri-Na–citrate, 30 mM glucose, pH 6.5). Diluted PRP was centrifuged at 69 *g* for 10 min at RT to pellet leukocytes. The supernatant was collected and centrifuged at 400 *g* for 10 min at RT to pellet the platelets. The supernatant was discarded and the platelet pellet was resuspended in 3 ml CGS buffer. Following an incubation of 5–10 min at RT, samples were centrifuged at 400 *g* for 10 min at RT and the supernatant was discarded. The platelet pellet was resuspended in HEPES buffer (145 mM NaCl, 5 mM KCl, 1 mM MgCl_2_, 10 mM glucose, 10 mM HEPES, pH 7.4) and the platelet concentration was adjusted to 2×10^9^ platelets ml^−1^. Platelets were incubated at 37°C in the water bath for 15 min. Platelet samples were frozen in liquid nitrogen for shock freeze lysis to obtain wPL and stored at −80°C.

### Bone substitute granules

We included six different CaPs in the experiments. Four kinds of granular, biphasic CaP (TCP/HA), with an intimate molecular mixture of 20% hydroxyapatite (HA) and 80% tricalcium phosphate (TCP), were kindly provided by Dr. Guy Daculsi (Biomatlante, France). The TCP/HA materials were synthesized and characterized according to the methods previously described in literature [26, 27]. The sample TCP/HA-1 was the conventional MBCP+ from Biomatlante (Biomatlante AMS group, CEmark and US FDA). The sample TCP/HA-2 was produced according to the same protocol with a higher sintering temperature to increase the crystal size and reduce microporosity [28]. The TCP/HA-3 sample was a smaller, rounded granule type with the same chemical composition and sintering as TCP/HA-1. The TCP/HA-4 sample had the same smaller size and rounded morphology as TCP/HA-3, but was produced with a higher sintering temperature as TCP/HA-2 [28].

Two kinds of granular β-TCP were purchased as chronOS Granules from DePuy Synthes, USA. No further modification was applied for the experiments. The characterization data for the β-TCP samples were retrieved from a previous study from Duan *et al*. [29], in which the values are linked to the sample ‘chronos’.

### Brunauer, Emmett and Teller measurement

The specific surface area (SSA) of the TCP/HA samples was determined by the Brunauer, Emmett and Teller (BET) method, which was already described in previous works [26, 28, 30]. The measurements were performed by nitrogen gas adsorption on Micromeritics 3-FLEX equipment. About 100 mg per sample was weighed and degassed in vacuum conditions with 10^−3^ mbar at 150°C for 24 h. Next, the weight of the samples was precisely measured. The SSA was calculated from the range of relative pressure of adsorption–desorption isotherms by 0.05–0.2. The unit of the SSA, determined by BET is in m^2^ g^−^^1^.

### Scanning electron microscopy

The morphology of the CaP materials was characterized by performing scanning electron microscopy (SEM) with a LEO 1530 GEMINI (Zeiss, Germany). The CaP surfaces were imaged in their native state at 3 kV and 121 V, without utilizing precious metal sputter coating.

### Protein adsorption preparation

Protein adsorption preparation was performed with modifications as previously described [31]. After thawing, human cP, PL and wPL were centrifuged at 20 000 *g* for 30 min at 4°C (5804R, Eppendorf, Germany) to remove protein aggregates and cellular debris. To ensure reproducibility, 10 mg of CaPs were used and incubated in either 1 ml cP or PL, or in 100 μl wPL. The samples were incubated for 1 h at 37°C. To deplete loosely bound or free protein, the CaPs were washed three times with PBS with centrifugation steps of 20 000 *g* for 10 min at 4°C, discarding the supernatant and adding 1 ml PBS to the granules. The washed and adsorbed proteins were desorbed by adding 50–100 μl of desorption buffer (2% (w/v) SDS, 62.5 mM Tris-HCl) to the CaPs, incubating the samples at 95°C for 5 min and centrifuging at 20 000 *g* for 10 min at 4°C. This protein desorption approach was performed and described in previous studies with different materials [31–33]. The supernatant was collected and analyzed further by protein quantification, sodium dodecyl sulfate-polyacrylamide gel electrophoresis (SDS-PAGE), ELISA, and LC–MS.

To utilize CaPs with washed and adsorbed proteins in tube formation assays, 10 mg CaP was added to 5 ml Medium 200 after the abovementioned protein adsorption preparation for a concentration of 2 mg ml^−1^. For the experiments with unwashed CaPs, CaPs were added directly to the supplemented medium for a concentration of 2 mg ml^−1^. Medium 200 was supplemented with 100 U ml^−1^ penicillin, 100 mg ml^−1^ streptomycin and with or without simple concentrated (1×) large vessel endothelial supplement (LVES; all Gibco, Germany) after washing steps.

### Protein quantification

Protein concentration of cP, PL, wPL and adsorbed protein samples was quantified with Pierce™ 660 nm Protein Assay Reagent (Thermo Scientific, Germany) according to the manufacturer’s instructions. For samples containing SDS, such as the desorbed proteins, Ionic Detergent Compatibility Reagent (Thermo Scientific, Germany) was added to the assay reagent, before employing the measurement. Bovine serum albumin (Sigma-Aldrich, Germany) was used as a standard. Absorption was measured by an Infinite M1000 plate reader (Tecan, Switzerland) at 660 nm.

### Sodium dodecyl sulfate-polyacrylamide gel electrophoresis

For cP, PL, wPL and adsorbed protein samples, 2 µg of protein was adjusted with deionized water to a total volume of 26 μl. To the diluted samples, 4 μl of NuPAGE™ Sample Reducing Agent and 10 μl of NuPAGE™ LDS Sample Buffer (both Invitrogen, Germany) were added and incubated at 70°C for 10 min to denature the proteins. The samples were loaded on a Bolt™ 10% Bis-Tris Plus gel using NuPAGE™ MES SDS Running Buffer (both Invitrogen, Germany) and run for 1 h at 200 V. SeeBlue™ Plus2 Pre-Stained Standard (Invitrogen, Germany) was used as a molecular weight marker. The gels were stained with SilverQuest™ Silver Staining Kit (Invitrogen, Germany) according to the manufacturer’s instructions and documented with the Scanning system View Pix 1100 (Biostep, Germany).

### Enzyme-linked immunosorbent assay

The concentration of human VEGF, human FGF-2 and human platelet-derived growth factor (PDGF-AB) was measured for cP, PL, wPL and wPL-adsorbed proteins samples by ELISA. Samples and reagents were prepared and processed according to the manufacturer’s instructions of the utilized Human VEGF Quantikine ELISA Kit, Human FGF2 Quantikine ELISA Kit and Human PDGF-AB Quantikine ELISA Kit (all R&D Systems, Germany). The absorption was measured by an Infinite M1000 plate reader at 450 nm with a wavelength correction at 570 nm.

### In solution digestion

Adsorbed protein samples were processed with Pierce™ Detergent Removal Spin Columns (Thermo Scientific, Germany) to remove SDS. The procedure was performed according to the manufacturer’s instructions before digestion. Subsequent protein digestion was carried out according to published protocols [32, 34]. For cP, PL, wPL and adsorbed protein samples, 25 μg was precipitated with ProteoExtract Protein Precipitation Kit (CalBioChem, Germany), following the manufacturer’s instructions. Afterwards, Proteins were isolated by centrifugation at 10 000 *g* for 10 min at RT and washed twice with ProteoExtract Protein Precipitation Washing Solution. The supernatant was discarded and the pellet left to dry for 5–10 min. Subsequently, the pellets were resuspended in 0.1% RapiGest SF surfactant (Waters Corporation, Germany), dissolved in 50 mM ammonium carbonate buffer. Samples were incubated at 80°C for 15 min to solubilize protein. Protein disulfide bonds were reduced by adding dithiothreitol (Sigma, Germany) solution in a final concentration of 5 mM. The reaction was performed at 56°C for 45 min. Next, proteins were alkylated by adding 500 mM iodoacetamide (Sigma, Germany) solution for a final concentration of 15 mM. The reaction was carried out in the dark for 1 h. Protein digestion was performed with a ratio of 50:1 of protein:trypsin (Promega, Germany) for 16 h at 37°C. The digestion was stopped by adding 2 μl of hydrochloric acid (Sigma, Germany) and incubating for 45 min at 37°C. Lastly, Samples were centrifuged at 13 000 *g* for 15 min to remove degradation products and the supernatant was transferred into new tubes.

### Liquid chromatography coupled to mass spectrometry analysis

The LC–MS measurements were performed with modifications from previously described methods [31]. Samples were diluted with LC–MS grade water (Merck, Germany) containing 0.1% formic acid (Sigma, Germany). Samples were spiked with 50 fmol μl^−1^ HI3 Ecoli Standard (Waters Corporation, Germany) for absolute protein quantification [35]. The digested peptides were applied to a nanoACQUITY UPLC system, equipped with a C18 nanoACQUITY trap column (5 μm, 180 μm × 20 mm) and a C18 analytical reversed-phase column (1.7 μm, 75 μm × 150 mm; all Waters Corporation, Germany). The two mobile phases consisted of (A) 0.1% (v/v) formic acid in water and 0.1% (v/v) formic acid in acetonitrile (Biosolve, Germany) and a gradient of 2–37% of mobile phase B over 70 min were used for separation. The samples were infused with a flow rate of 0.3 μl min^−1^ and the referent components Glu-Fibrinopeptide and LE (both Sigma, Germany) were set to a flow rate of 0.5 μl min^−1^. The nanoACQUITY UPLC system was coupled with a Synapt G2-Si mass spectrometer (Waters Corporation, Germany). Electrospray ionization (ESI) was performed with a NanoLockSpray source was in positive mode. The measurements were conducted in resolution mode and experiments were carried out with data-independent acquisition (MS^E^). Each measurement was performed in technical triplicates.

A mass to charge range of 50–2000 Da, scan time of 1 s, ramped trap collision energy from 20 to 40 V was set and data were acquired over 90 min. For data acquisition and processing, the software MassLynx 4.1 (Waters Corporation) was utilized.

### Protein identification

Peptides and proteins were identified with the software Progenesis QI 2.0 (Nonlinear Dynamics) [31]. Noise reduction threshold for low energy, elevated energy and peptide intensity were set to 120, 25, and 750 counts, respectively. A human data base with reviewed proteins was downloaded from uniport (SWISS PROT) and modified with the sequence information of Hi3 E. coli standard for absolute quantification. Following parameters were selected: one missed cleavage, maximum protein mass 600 kDa, fixed carbamidomethyl modification for cysteine and variable oxidation for methionine. Protein identification requirements were restricted to at least two assigned peptides and five assigned fragments. Peptide identification requirements were based on three assigned fragments. Identified peptides with a score parameter below 4 were excluded. Protein amount in fmol was calculated by TOP3/Hi3 approach [36]. An overview of all identified proteins is accessible in separate Supplementary Information.

### Human umbilical vein endothelial cell culture

Human umbilical vein endothelial cells (HUVECs) (Gibco, Germany) were cultured with Medium 200, which was supplemented with simple concentrated (1×) LVES, 100 U ml^−1^ penicillin and 100 mg ml^−1^ streptomycin. The cells were kept in an incubator at 37°C, 5% CO_2_ and 95% relative humidity (CO_2_ Incubator C200, Labotect, Germany) for cultivation. For cell subculture and harvesting for experiments, cells were briefly washed with PBS, followed by cell detachment with 0.25% Trypsin–EDTA (Gibco, Germany) for 5 min at 37°C, 5% CO_2_ and 95% relative humidity. The cell suspension was transferred with LVES-supplemented medium and centrifuged at 130 *g* for 5 min at RT (5810R, Eppendorf, Germany). The supernatant was discarded and the cell pellet resuspended in LVES-supplemented medium. Cell viability and cell count were determined by equally mixing 20 µl of cell suspension and trypan blue and measuring by an automated cell counter (TC10, Bio-Rad, Germany).

### Tube formation assay

Geltrex™ LDEV-Free Reduced Growth Factor Basement Membrane Matrix (Gibco, Germany) was thawed on ice at 4°C overnight. The wells of a μ-Slide Angiogenesis (Ibidi, Germany) were covered with 10 μl of basement membrane matrix and incubated at 37°C, 5% CO_2_ and 95% relative humidity for 1 h to solidify. HUVECs with a passage number between 2 and 6 were harvested and centrifuged as described above. The pellet was resuspended in Medium 200 without LVES to avoid undesired stimulation. The cell concentration was adjusted to 400 000 cell ml^−1^ and mixed with the same volume of supplemented medium for each test condition, respectively. A cell number of 10 000 cells per well was seeded. The cells were incubated at 37°C, 5% CO_2_ and 95% relative humidity and examined for tube formation after 18 h under a CKX41 inverted microscope (Olympus, Germany). One picture was taken per well by utilizing the software analySIS getIT (Olympus Soft Imaging Solutions GmbH). Pictures were evaluated with ImageJ (National Institutes of Health, USA) and the plugin Angiogenesis Analyzer (Gilles Carpentier) [37], measuring the total segment length.

### Data representation

Data were presented as means ± standard deviation (SD) of the values. Statistical analysis was performed with GraphPad Prism 7 (GraphPad Software, USA). For a comparison of two data sets, the unpaired *t-*test was utilized. For statistical analysis, involving multiple data sets, one-way analysis of variance (ANOVA) with Tukey’s multiple comparison test or Dunnett’s multiple comparison test were performed. All tests were carried out choosing a *P* values of <0.05 to be statistically significant.

## Results

**Figure 1.**
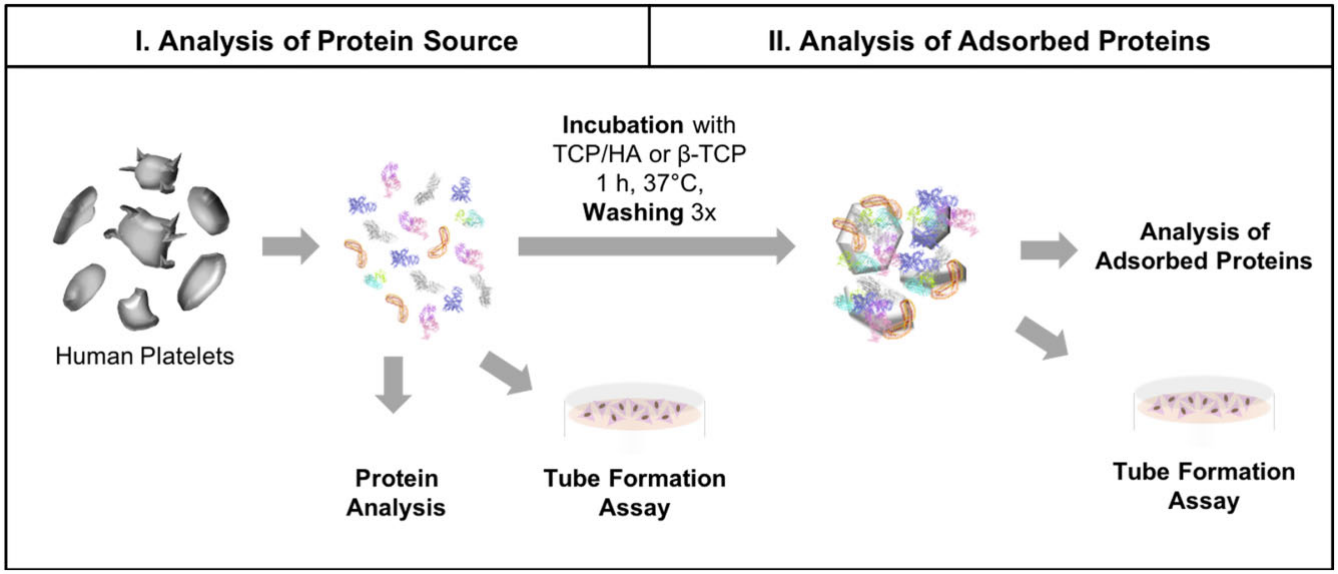
Principal workflow of protein source and adsorbed protein analysis. PLs were generated from human platelets in plasma and from washed platelets, respectively. Different bone substitutes were incubated with hemoderivative protein sources for protein adsorption. Incubated bone substitutes were washed three times with PBS, pH 7.4 to remove loosely bound and free proteins. Afterward, adsorbed proteins were recovered by a protein desorption step. Protein sources and adsorbed protein samples were analyzed by protein quantification, SDS-PAGE, silver staining and LC–MS. Tube formation assays were employed to study pro-angiogenic effects of hemoderivative protein sources and bone substitutes with adsorbed proteins. Protein crystal structures were taken from RCSB protein data bank. Images are not drawn to scale.

We investigated lysates of platelet with plasma (PL) and from washed platelets (wPL) in a divided workflow with two steps (Fig. 1). Additionally, we included human citrate plasma (cP) was to compare the PL preparations with plasma and without. These three hemoderivative protein sources (cP, PL and wPL) were used to study protein adsorption on CaP surfaces. The hemoderivative protein sources and adsorbed protein samples were analyzed qualitatively by SDS-PAGE and by LC–MS for quantitative proteomic data. In addition, the samples were analyzed for selected pro-angiogenic growth factors by ELISA. Finally, the pro-angiogenic potential of the samples was evaluated by employing tube formation assays.

### Hemoderivative protein sources

**Figure 2.**
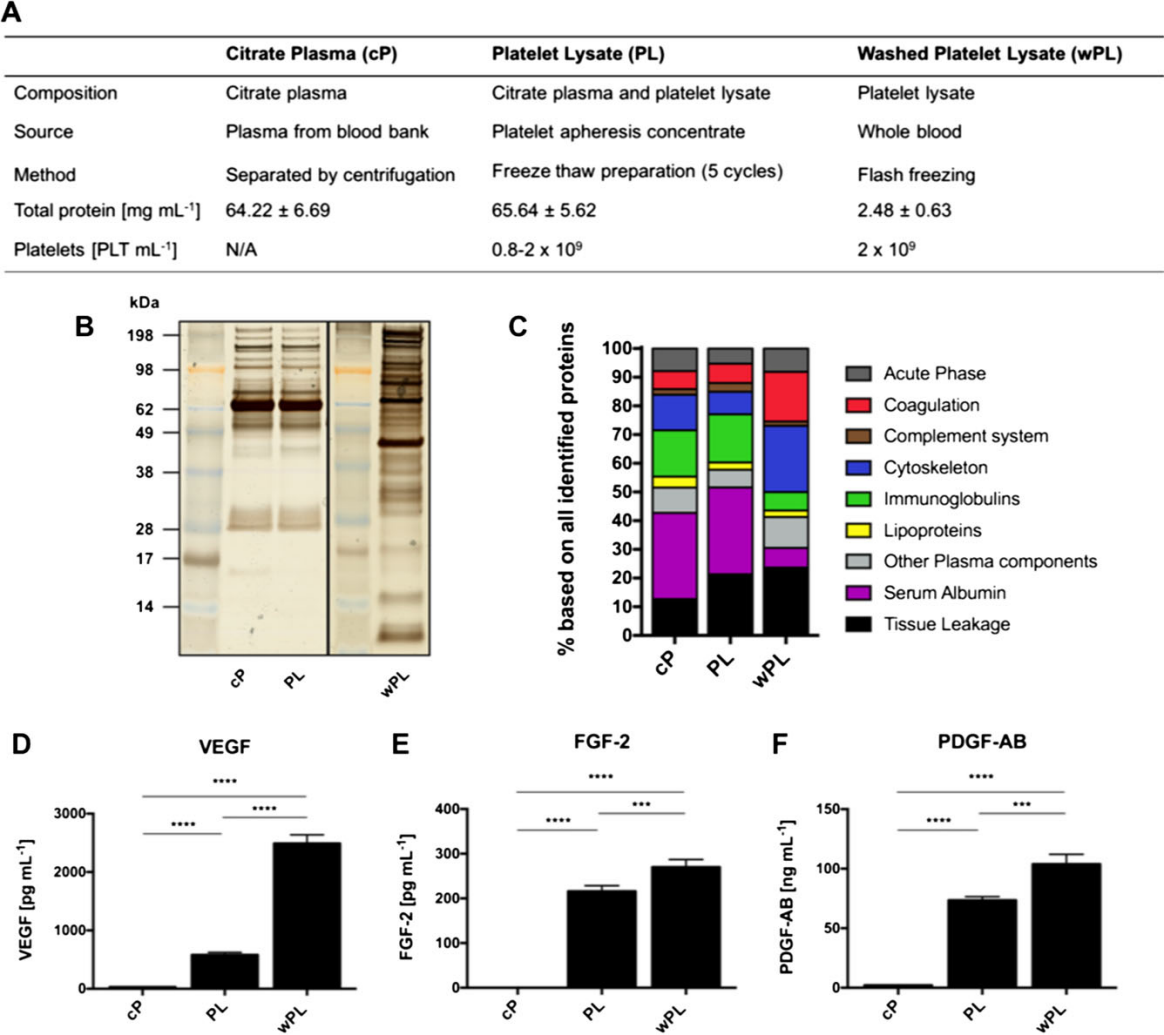
Human washed PL differs from plasma and PL in protein composition and growth factor concentration. (**A**) Characteristics of utilized hemoderivative protein sources. The main composition, source of material, preparation method, protein concentration (mg ml^−1^ ± SD, *n* = 4–8) and platelet (PLT) count are listed. Protein sources were analyzed by (**B**) SDS-PAGE with silver staining and (**C**) quantitative LC–MS proteomics. The bars indicate the percentage based on all identified proteins with protein annotation in nine different functional classes. (**D**–**F**) Protein sources were evaluated for pro-angiogenic growth factors. The concentration of VEGF, FGF-2 and PDGF-AB was measured by ELISA (data are shown as mean ± SD, *n* (PDGF-AB, cP) = 2, *n* (others) = 4). The statistical significance was calculated by ANOVA with Tukey’s multiple comparison test (***P < 0.001, ****P < 0.0001); N/a, data not available.

The three hemoderivative protein sources used in this study were distinct in composition and source material (Fig. 2A). Human cP was obtained from the blood bank. The first type of human PL was generated from human platelet apheresis concentrates by a freeze–thaw preparation of five cycles. The human wPL was generated from whole blood of single healthy human donors. Here, the platelets were washed to remove plasma components before lysis. Both lysates were generated with a similar platelet concentration. However, wPL showed a lower protein concentration when compared with PL, indicating the depletion of plasma proteins.

The plasma protein depletion was also demonstrated by SDS-PAGE, observing a distinct band pattern for wPL compared with the other two sources (Fig. 2B). To further detail the differences of the complex protein composition between the hemoderivatives we performed quantitative LC–MS measurements. LC–MS-identified proteins of the three sources were assigned to nine different protein function classes (Fig. 2C). Here, cytoskeletal and coagulation-related proteins were more pronounced in wPL when compared with PL and cP and tissue leakage proteins were more pronounced in wPL when compared with cP. Conversely, a lower amount of immunoglobulins and serum albumin was detected in wPL. Minor differences were uncovered for PL in contrast to cP like a higher percentage amount of tissue leakage proteins, indicating lysed platelets. PL proteins, such as platelet factor 4 and platelet basic protein were also found within the ten most abundantly detected proteins for wPL, whereas plasma proteins, such as serum albumin and immunoglobulins were among the most abundant in cP and PL (Supplementary Fig. S1).

To provide data for pro-angiogenic growth factors, the concentration of VEGF, fibrinogen growth factor 2 (FGF 2) and platelet derived growth factor (PDGF-AB) were measured by ELISA (Fig. 2D–F). Here, both, PL and wPL showed a statistically significant higher concentration of all three growth factors when compared with cP. Comparing PL and wPL, the highest concentration for all three pro-angiogenic growth factors was found in wPL. However, as wPL was generated from single-donor blood, the concentration of all three growth factors showed donor-dependent differences (Supplementary Fig. S2).

**Figure 3.**
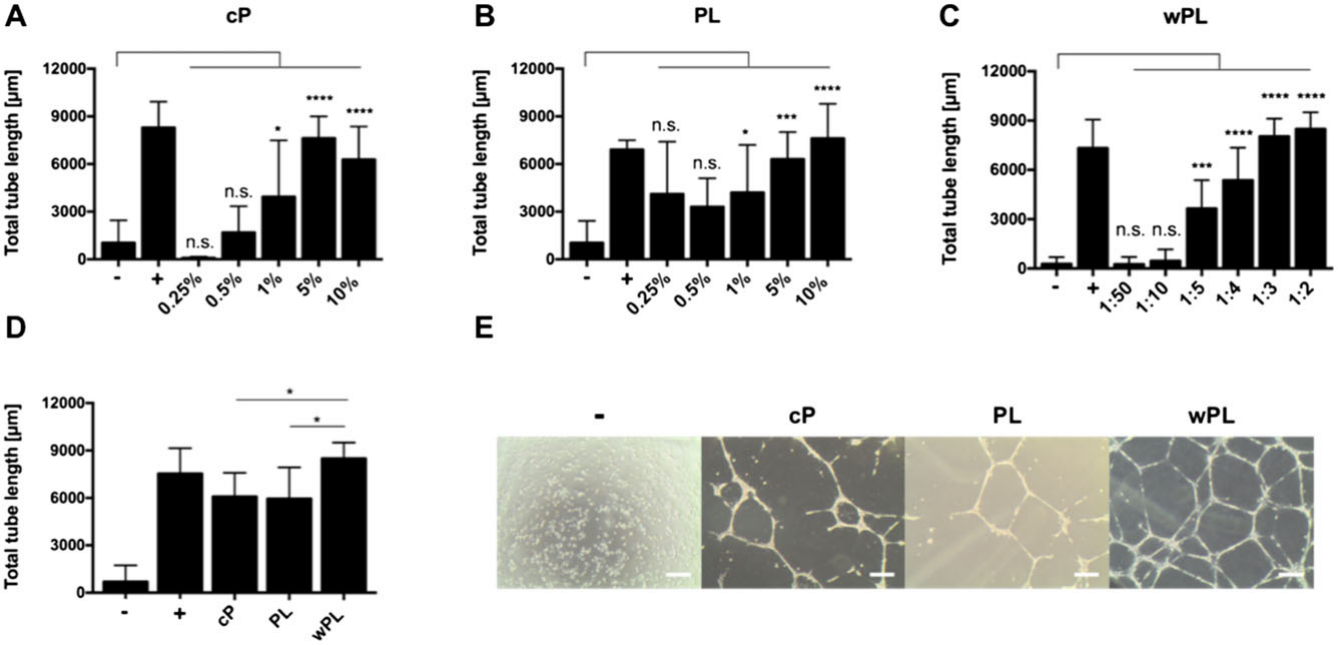
Human wPL induces stronger angiogenic response in tube formation assays. (**A**–**C**) The pro-angiogenic effect of hemoderivative protein sources was analyzed by employing tube formation assays with HUVECs. HUVECs were incubated with different concentrations of citrate plasma (cP), platelet lysate in plasma (PL) and lysate of washed platelets (wPL) on GeltrexTM LDEV-Free reduced growth factor basement membrane matrix for 18 h. For cP and PL different percentual dilutions (0.25–10%) in the cell culture medium were tested. For wPL different ratios of wPL in total volume (1:50–1:2) with cell culture medium were tested. Total tube length was evaluated with ImageJ and the plugin angiogenesis analyzer (−, negative control: medium without LVES; +, positive control: medium with LVES; data are shown as mean ± SD, *n* = 3–8). (**D**) Tube formation was analyzed with a comparable protein concentration of 1.1 mg/ml^−1^ for all three protein sources (data are shown as mean ± SD, *n* = 6–17). The statistical significance was calculated by ANOVA with Dunnett’s multiple comparison test (*P < 0.05, **P < 0.01, ***P < 0.001, ****P < 0.0001), performing the comparison with the negative control (A–C) or with wPL (D). (**E**) HUVECs on GeltrexTM after 18 h incubation with different hemoderivative protein sources at the same concentration, as seen in (**D**). The scale bars represent 200 µm.

In addition, we performed tube formation assays to study the pro-angiogenic potential and complement the protein data. Alongside the tested samples, we included non-supplemented cell medium as negative control (−) and LVES supplemented medium as positive, tube stimulating, control (+). Generally, increasing the concentration of all three hemoderivatives, respectively, increased the tube formation with statistical significance (Fig. 3A–C). Yet, a peak in total tube length was observed for cP at 5%, achieving slightly better results than 10% (Fig. 3A). Comparing the three hemoderivative sources at the same protein concentration of 1.1 mg/ml^−1^ revealed wPL to be the most potent inducer of tube formation (Fig. 3D and E). The enhanced tube formation by wPL could be observed microscopically and evaluated by the Angiogenesis Analyzer Plug-In of ImageJ, resulting in a statistically significant result. Comparable to the donor-dependent differences in growth factor concentration, the effect of the tube formation assays differed among the various donor samples (Supplementary Fig. S3).

### Protein adsorption

Protein adsorption influences the interplay between biomaterial surfaces and their biological environment. In this part, we investigated the protein adsorption of the three abovementioned and characterized protein sources on different CaP surfaces. Additionally, we measured the concentration of the same growth factors and linked the protein results to the results of the tube formation assays.

**Figure 4.**
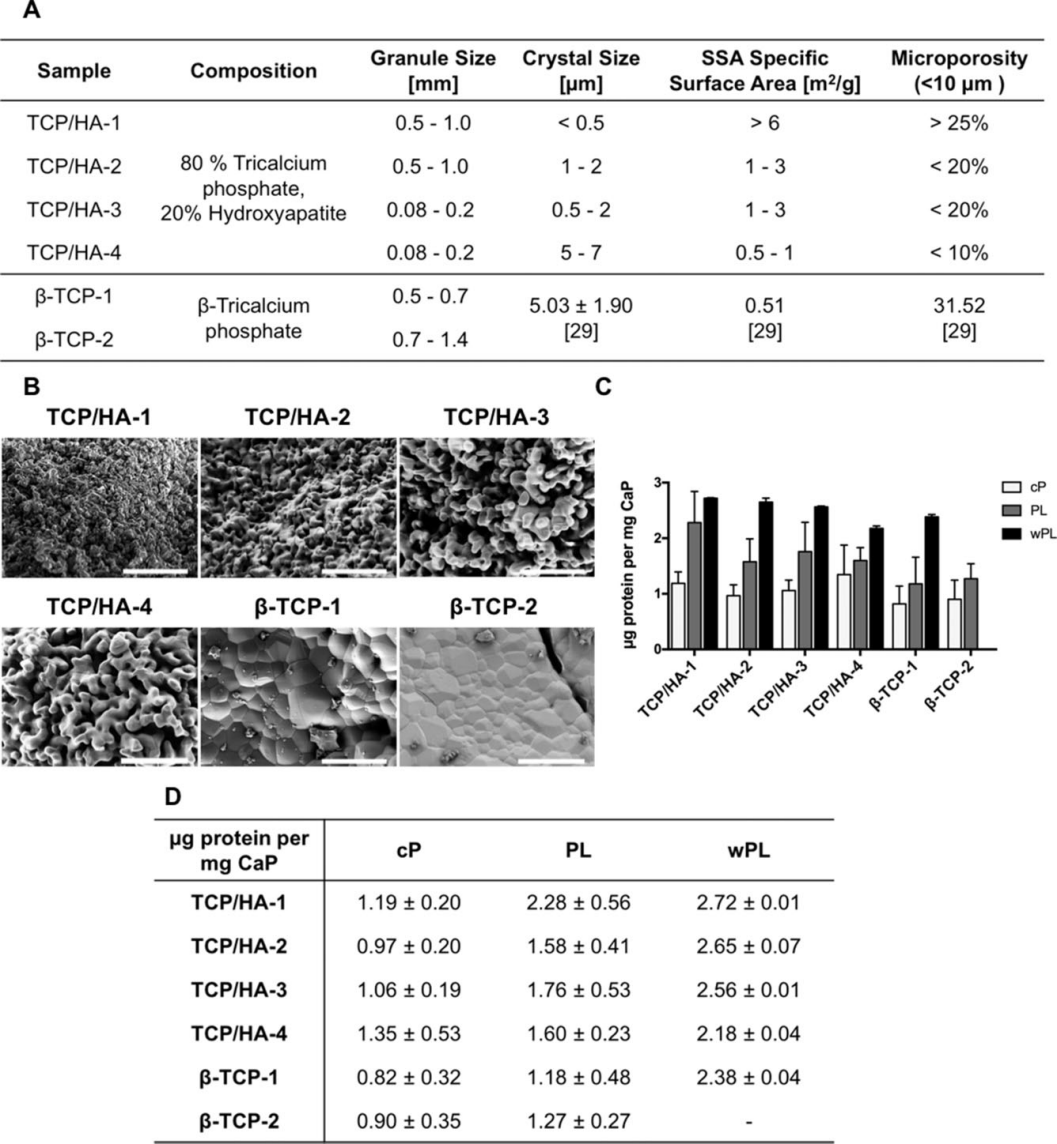
CaP Material characterization, SEM images and protein adsorption. (**A**) The physicochemical characterization of utilized CaP materials. The composition, size of the granules, crystal size, SSA, microporosity and the manufacturing processes are listed. Crystal size, SSA and microporosity for β-TCP materials were retrieved from previously published measurements [29]. (**B**) SEM images of granular CaP surfaces. The scale bars represent 10 µm. (**C**) Quantification of adsorbed proteins on CaPs, shown as a bar diagram. CaPs were incubated for 1 h in cP, PL and wPL, respectively. Protein adsorption of wPL on β-TCP 2 was not performed and analyzed due to limited quantities of wPL and strong similarities between β-TCP 1 and β-TCP 2 for cP and PL. Subsequently, three wash steps were performed and proteins were desorbed with 2% SDS. Proteins were quantified by Pierce 660 nm Assay (data are shown as mean ± SD, *n* = 2–7). (**D**) Quantification of adsorbed proteins on CaPs, shown as a table, as seen in (C) (data are shown as mean ± SD, *n* = 2–7).

Four different CaP surfaces consisting of 80% TCP and 20% HA (HA/TCP) with two different granule sizes were selected. Additionally, we included two different CaP surfaces consisting of β-TCP with two different sizes, which were commercially purchased as chronOS (DePuy Synthes). The physicochemical characterization revealed differences between the materials (Fig. 4A and B; Supplementary Fig. S4). The HA/TCP samples showed an increasing crystal size with a raising sample number. The β-TCP samples showed the highest crystal size with HA/TCP-4. Conversely, the SSA decreased within HA/TCP a raising sample number, and the β-TCP samples showed the smallest SSA with HA/TCP-4. The microporosity decreased within HA/TCP sample number. The microporosity of β-TCP was comparable to the higher microporosity of HA/TCP-1 (Fig. 4A).

To investigate the protein adsorption, we incubated the CaP materials with the hemoderivatives for 1 h at 37°C and subsequently washed them three times before a desorption step. This protein desorption approach was performed and described in previous studies with different materials [31–33]. Proteins were detected for all material variations incubated in all three hemoderivative sources. We did not perform and analyze protein adsorption of wPL on β-TCP-2 due to limited quantities of wPL and great similarities between β-TCP-1 and β-TCP-2 in the case of adsorption with cP and PL. Interestingly, the highest concentration of adsorbed proteins was consistently measured for all materials after being incubated in wPL when compared with the other sources (Fig. 4C and D). For cP, we observed similar amounts of protein regardless of the used CaP surface. On the other hand, we noticed a decreasing trend of protein amount in the case of PL, which was following the decreasing SSA for the materials, reversibly the increasing crystal size. This same trend was observed for wPL in the case of the TCP/HA samples.

**Figure 5.**
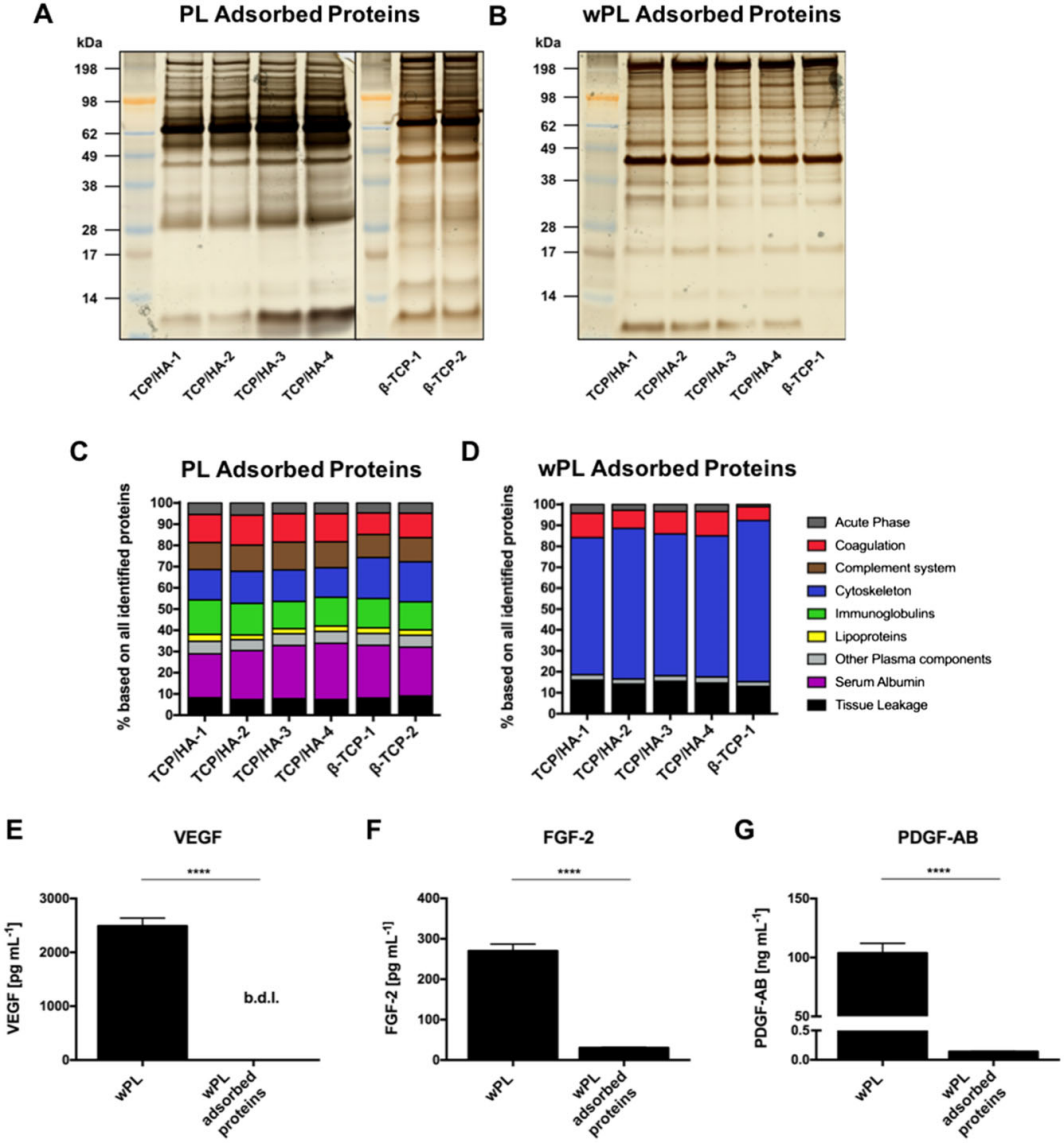
Cytoskeletal proteins from human washed PL are enriched on CaP surfaces while pro-angiogenic growth factors are depleted. CaP surfaces were incubated for 1 h in the hemoderivative protein source. After three subsequent washing steps, proteins were desorbed with 2% SDS. (**A**, **B**) SDS-PAGE and silver staining were performed for adsorbed proteins on different CaP surfaces incubated in PL and wPL, respectively. Protein adsorption of wPL on β-TCP 2 was not performed and analyzed due to limited quantities of wPL and strong similarities between β-TCP 1 and β-TCP 2 for cP and PL. (**C**, **D**) Adsorbed proteins on CaP surfaces from PL and wPL, respectively, were analyzed by quantitative LC–MS proteomics and identified proteins were classified into nine different protein functional classes. The bars indicate the percentage based on all identified proteins. (**F**–**G**) Adsorbed proteins on the CaP surface of TCP/HA-4, incubated in wPL, were evaluated for angiogenesis-involved growth factors and compared with wPL. The concentration of VEGF, FGF-2 and PDGF-AB was measured by ELISA (b.d.l. = below detection limit; data are shown as mean ± SD, *n* = 4). The statistical significance was calculated by an unpaired *t* test (****P < 0.0001).

The band pattern of the adsorbed proteins, as seen on SDS-PAGE, differed slightly from their original hemoderivative protein sources’ pattern. The protein pattern complexity was slightly reduced for wPL-related protein adsorption compared with cP and PL, respectively (Fig. 5A and B; Supplementary Fig. S5A). Differences were more pronounced when the adsorbed proteins from wPL were compared with the absorbed proteins from cP and PL. Both cP and PL showed their strongest band at ∼66 kDa, suggesting the rough molecular weight of serum albumin [38]. The strongest band for wPL was defined at ∼42 kDa, suggesting β-actin [38, 39]. Interestingly, the band pattern was similar across the different materials when incubated in the same hemoderivative source. Only slight differences were observed on the gel between TCP/HA materials and β-TCP.

**Figure 6.**
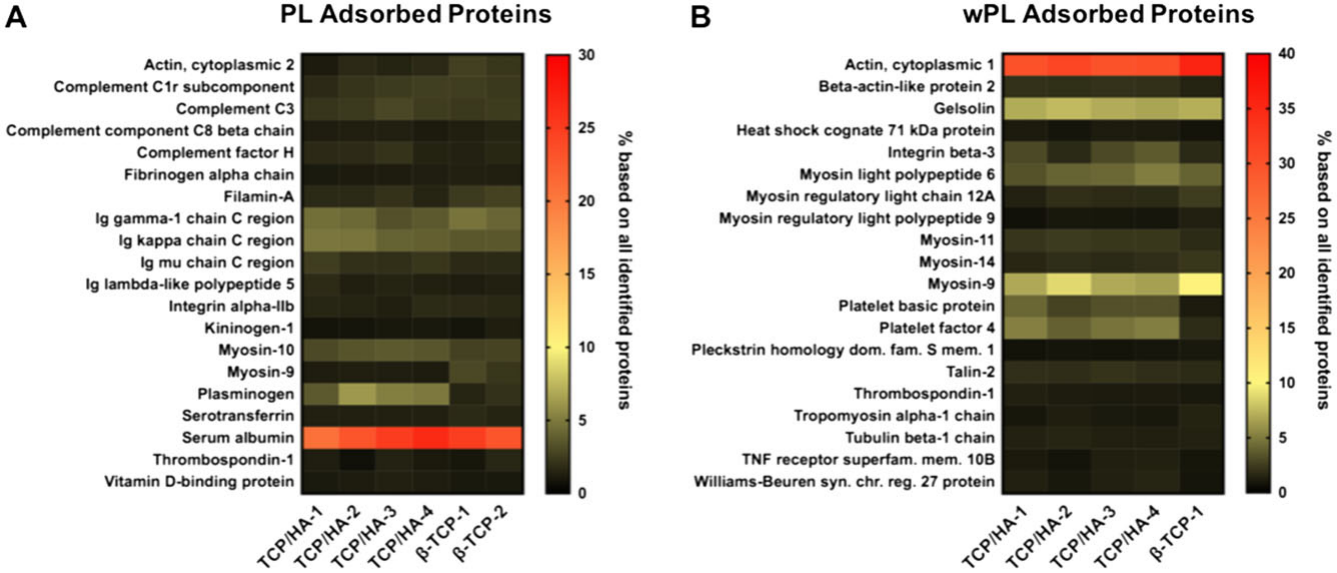
Non-regenerative and anti-angiogenic proteins are detected among the most abundant proteins on PL incubated CaP. (**A**, **B**) The heat maps show the 20 most abundant proteins for TCP/HA-4 compared with the other CaP surfaces. The values represent the percentage based on all identified proteins.

We employed LC–MS measurements to further characterize the complex adsorbed protein composition. Using the same protein functional classes as shown for the hemoderivatives, large differences in adsorbed proteins were revealed depending on the utilized hemoderivative. After the incubation in cP, the most abundant protein was serum albumin, followed by immunoglobulins (Supplementary Fig. S5B). Likewise, serum albumin remained the highest protein in PL, although decreased. The other protein functional classes in PL, such as coagulation, complement, cytoskeleton and immunoglobulins were distributed more evenly (Fig. 5C). The protein functional class distribution of wPL was quite distinct compared with cP and PL. Here, cytoskeleton proteins were found to be the most abundant group with more than 60% on every CaP surface used, followed by tissue-leakage proteins and coagulation (Fig. 5D). Generally, the protein composition by protein functional classes did not differ much between the different CaP materials within the same adsorbed protein source.

Next, we quantified the three previously described pro-angiogenic growth factors by ELISA in the adsorbed protein samples and compared them with the original hemoderivative. The ELISA experiment was exclusively performed for TCP/HA-4, incubated in wPL. We chose to investigate TCP/HA-4 principally because of the small size of the granules (Fig. 4A) and therefore its suitability for the tube formation assays. We chose wPL as the hemoderivative for its best performance in the tube formation assays (Fig. 3D and E). Despite the promisingly high concentration of VEGF, FGF-2 and PDGF-AB in wPL, a great decrease was measured after protein adsorption (Fig. 5E–G). The value for VEGF was measured below the detection limit. FGF-2 was depleted ∼10-fold and PDGF-AB ∼700-fold. Additionally, single non-angiogenic and anti-angiogenic proteins were found among the 20 most abundant proteins in both PL and wPL-adsorbed protein samples. In case of the adsorbed proteins from PL, TSP-1 was found to be present among the top20 proteins. An anti-angiogenic potential of TSP-1 has been previously reported [40]. The other proteins detected were mainly non-angiogenic plasma proteins, such as albumin, immunoglobulin chains, and complement factors (Fig. 6A). For the adsorption of wPL proteins, the highest abundant proteins were found to be cytoskeleton proteins or cytoskeleton-associated proteins. Among these proteins were cytoplasmatic actin, myosin-family proteins, tubulin and gelosin, an actin-binding protein. Platelet prominent proteins detected were platelet basic protein, a precursor of the pro-angiogenic chemokines connective tissue-activating peptide III (CTAP-3) and neutrophil-activating peptide-2 (NAP-2), TSP-1 and PF4, a chemokine involved in anti-angiogenic signaling (Fig. 6B) [41, 42]. In comparison, the most abundantly adsorbed proteins after incubation in cP were serum albumin and several immunoglobulin chains, apolipoproteins and other prominent plasma proteins (Supplementary Fig. S5C). Here, the composition of the abundant proteins was found to be highly influenced by the presence of plasma proteins. In summary, non-angiogenic cytoskeleton proteins and anti-angiogenic platelet proteins adsorbed on the CaP surfaces and the inclusion of plasma drastically changes the adsorbed proteins.

**Figure 7.**
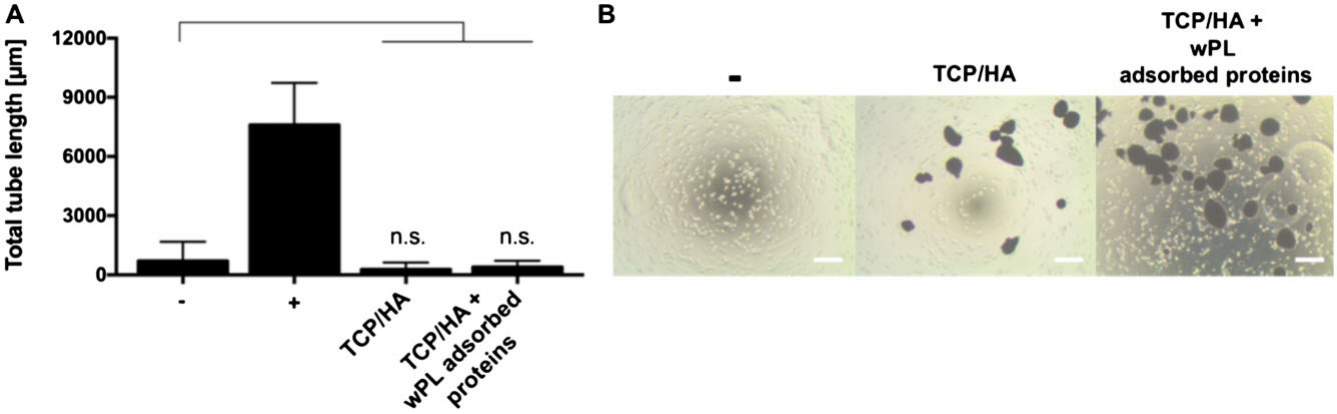
Washed CaP surfaces incubated in human washed PL show no pro-angiogenic effect. (**A**) The pro-angiogenic effect was analyzed by employing tube formation assays with HUVECs. HUVECs were incubated with untreated CaP surfaces and CaP surfaces, incubated in wPL and washed three times (all TCP/HA-4). HUVECs were then seeded on GeltrexTM LDEV-Free reduced growth factor basement membrane matrix for 18 h. Total tube length was evaluated with ImageJ and the plugin angiogenesis analyzer (−, negative control: medium without LVES; +, positive control: medium with LVES; data are shown as mean ± SD, *n* = 4–9). The statistical significance was calculated by ANOVA with Dunnett’s multiple comparison test (*P < 0.05), performing the comparison with the negative control. (B) HUVECs on GeltrexTM after 18 h incubation with untreated CaP surfaces and CaP surfaces (seen as dark spots in the images), incubated in wPL and washed three times, as seen in (A). The scale bars represent 200 µm.

Subsequently, the selected CaP material, TCP/HA-4 was tested without and with preincubation in wPL in tube formation assays to investigate the effects on the angiogenic performance of HUVECs. While wPL potently induced tube formation, no angiogenic effect was observed when preincubated with TCP/HA and then washed with PBS. In this case, we obtained the same negative result for non-treated CaP materials and wPL preincubated CaP materials (Fig. 7).

**Figure 8.**
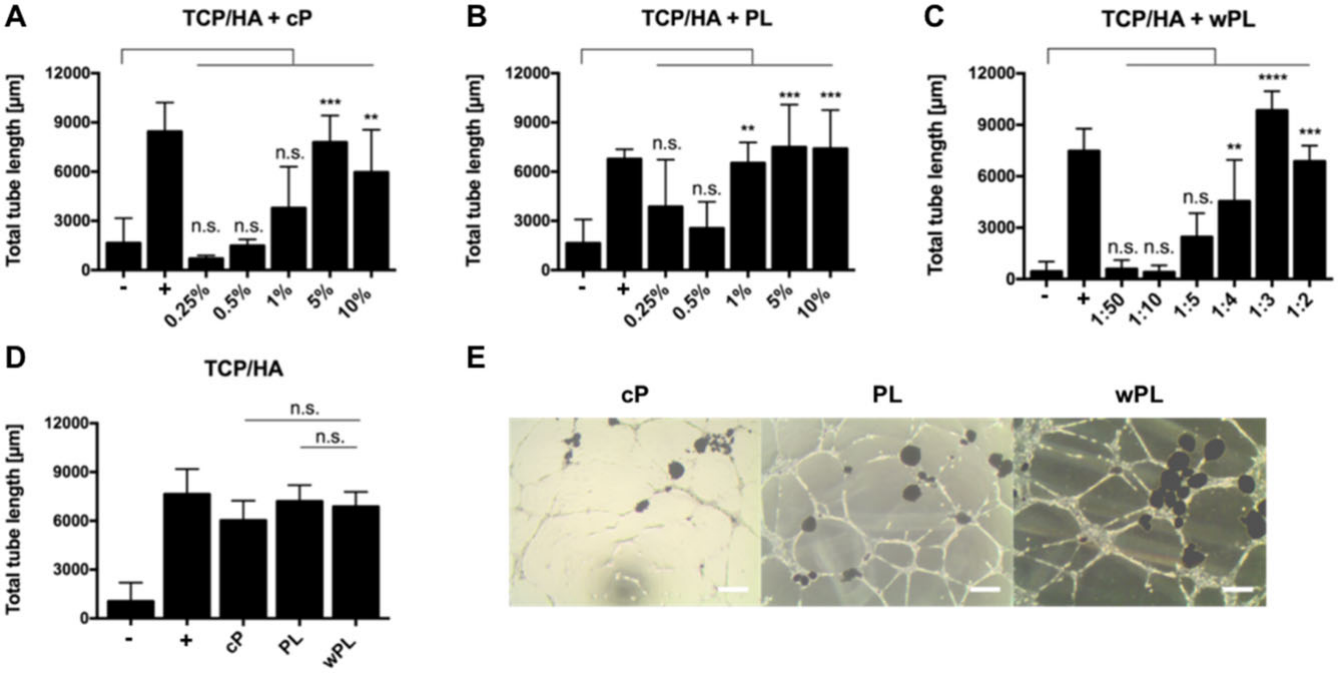
CaP Surfaces kept in different hemoderivative protein source show a similar pro-angiogenic stimulation. (**A**–**C**) The pro-angiogenic effect of CaP surfaces in hemoderivative protein sources was analyzed by employing tube formation assays with HUVECs. HUVECs were incubated with different concentrations of cP, PL and wPL, containing CaP on GeltrexTM LDEV-Free reduced growth factor basement membrane matrix for 18 h (all TCP/HA-4). for cP and PL different percentual dilutions (0.25–10%) in the cell culture medium were tested. For wPL different ratios of wPL in total volume (1:50–1:2) with cell culture medium were tested. Total tube length was evaluated with ImageJ and the plugin angiogenesis analyzer (−, negative control: medium without LVES; +, positive control: medium with LVES; data are shown as mean ± SD, *n* = 3–6). (**D**) Tube formation was analyzed with the same protein concentration of 1.1 mg/ml for all three protein sources, containing CaP without additional washing with PBS (all TCP/HA-4, data are shown as mean ± SD, *n* = 6–17). The statistical significance was calculated by ANOVA with Dunnett’s multiple comparison test (*P < 0.05, **P < 0.01, ***P < 0.001, ****P < 0.0001), performing the comparison with the negative control (A–C) or with wPL (D). (**E**) HUVECs on GeltrexTM after 18 h incubation with different hemoderivative protein sources at the same concentration, containing CaP (seen as dark spots in the images), as shown in (D). The scale bars represent 200 µm.

Additional tube formation assays were performed with TCP/HA-4 in the presence of the three respective hemoderivatives, but without removing the hemoderivatives and washing with PBS after adding TCP/HA-4 to the hemoderivatives. For the three hemoderivatives, concentration-dependent effects on the tube formation were observed, similar to the experiments without CaP surfaces. However, CaP surfaces incubated in both, CP and wPL seemed to induce less tube formation when incubated in the lowest dilution, 10% and 1:2, respectively (Fig. 8A–C). Also, similar protein concentrations of the hemoderivative were tested. Unlike the results for hemoderivative sources without CaP surfaces, all three hemoderivatives induced tube formation, but without statistical difference between the hemoderivative groups (Fig. 8D). However, the tube formation efficiency tended to be visually stronger for the conditions with PL and wPL compared with cP. Here, the HUVECs attached to the CaP surfaces and formed tubes with the CaP surfaces as central nodes (Fig. 8E).

## Discussion

In this study, we investigated comparatively cP (citrate plasma), PL (lysate of platelets in plasma) and wPL (lysates of washed platelets without plasma) and their effect as sources of pro-angiogenic factors adsorbed on CaP surfaces. Despite the difference in composition of the three hemoderivative sources, all of these promoted tube formation of HUVECs, which is in accordance with other reports [43–45]. However, the concentration of the prominent growth factors VEGF, FGF-2 and PDGF-AB was higher in the platelet-derived sources, PL and wPL than in plasma alone. Depleting plasma components from isolated platelets by washing resulted in both, an elevated concentration of growth factors and improved tube formation, indicating the effectiveness of lysates, such as wPL for regenerative effects. Nevertheless, it has to be considered that non-pooled wPL show inter-individual differences in the experiments, which might have an impact on the use of autologous donor materials to promote regenerative effects.

Using the same workflow, including mass spectrometry-based proteomic analysis, we investigated the complex protein composition of the hemoderivatives adsorbed on different CaP materials, which revealed significant differences. Despite differences in the physicochemical properties of the CaP surfaces, we quantified comparable amounts of protein per amount of CaP in the case of cP and wPL but observed a decrease in protein amount with a decrease of the material’s SSA for adsorbed PL and wPL. For the latter protein source, the trend was observed for the TCP/HA samples. The impact of the SSA on protein adsorption was described in the literature [29, 46]. In any case, the protein adsorption on biomaterials represents a highly complex process that is driven by multiple factors and, therefore, challenging to predict. As described through experimental observations, adsorption on CaPs is influenced on one hand by the material’s physicochemical properties such as the surface area, the chemical composition, hydrophobicity and the topography. On the other hand, the proteins’ properties, such as size, charge and structure add a secondary dimension of influence [2, 47]. Complex protein mixtures as the herein described hemoderivative protein sources can therefore bring a variation to the adsorption result. Despite having a lower SSA, β-TCP-1 adsorbed more protein from wPL than the TCP/HA samples with a higher SSA. Seemingly, the higher microporosity and the chemical composition influence this result. For the cP-related adsorption, it is necessary to point out that plasma proteins are vastly different from lysate-derived proteins. While both lysates contain a significant amount of cellular components, such as membrane proteins, lipids and metabolites, plasma is comprised mainly of secreted and globular proteins. This difference in composition results in similar protein amounts adsorbed throughout all CaP samples despite their physicochemical features. Similar observations were made in a study when incubating various CaP in serum [48]. Accordingly, the biomaterial's physicochemical properties must be considered together with the composition of the protein source to evaluate the performance of protein adsorption.

Increased adsorption of cytoskeleton proteins was observed when PLs, especially from wPL, were incubated with the CaP materials. Among the most 20 abundant platelet proteins, non-regenerative proteins, such as cytoskeleton proteins, and anti-angiogenic proteins, such as PF4 and TSP-1 were identified. These proteins are abundantly expressed in human platelets with estimated copy numbers of 563 000 and 101 000 per platelet, respectively [49]. PF4 and TSP-1 inhibit the proliferation and migration of endothelial cells and induce apoptosis via multiple mechanisms [50, 51]. One major anti-angiogenic effect of PF4 on endothelial cells is its ligation of the important growth factors VEGF and FGF-2, thereby preventing their endothelial interaction, especially through its competitive binding to heparin and heparin sulfate of proteoglycan receptors [52, 53]. Similar to PF4, TSP-1 also interferes with endothelial cell binding of VEGF. In addition, TSP-1 signals via CD47, which is coupled to the VEGF receptor R2 to reduce VEGF-mediated activation and which counteracts nitric oxide signaling in endothelial cells [54, 55]. However, there are also studies reporting pro-angiogenic effects of TSP-1 via indirect signaling through myofibroblasts [40, 56]. Furthermore, a recent study demonstrated a pro-angiogenic potential of TSP-1 for biomaterial surface modification [57]. Future studies must clarify the role of TSP-1 as a stimulant for pro-angiogenic biomaterial modification. CXCL7 is the most abundant platelet chemokine stored in the α-granules. Platelet basic protein represents a precursor CXCL7 variant of the pro-angiogenic active forms CTAP-III and NAP-2 [42] with an estimated copy number of 479 000 per platelet [49]. It has been shown that the proteolytic cleavage product of platelet basic protein, CTAP-III mediates chemotaxis of endothelial cells *in vitro* [58]. However, NAP-2 showed the most chemotactically regulatory activity on neutrophils [42]. Platelet basic protein exerts antimicrobial activity, but its effect on endothelial cells is unknown so far [59].

The observed lacking pro-angiogenic response of HUVECs after incubation with CaP materials adsorbed with proteins from PLs might be due to a decreased binding affinity of the growth factors VEGF, FGF-2, PDGF-AB and increased binding affinity of the anti-angiogenic protein platelet factor 4 for the CaP materials, which were washed with PBS. Potentially, this washing procedure in the absence of divalent cations, e.g. Ca2+ and Mg2+ ions, may affect the types and amount of adsorbed proteins as non-washed CaP surfaces kept in hemoderivative protein source, still induced angiogenesis of cultured HUVECs. However, here all three tested plasma and platelet protein sources showed similar pro-angiogenic effects compared with the positive control (LVES-supplemented medium). Additionally, we observed an inclusion of the CaPs in the HUVEC’s tubular structures, demonstrating an interaction between the cells and the CaPs. This adhesion of HUVECs on CaPs was already described in the literature [60]. It can be assumed that integrins on HUVECs establish contact with several adsorbed proteins with integrin-binding domains on the CaPs, such as fibronectin or vitronectin. This formation of a focal contact between CaPs and cells through protein contacts was experimentally explained for marrow stromal cells, osteosarcoma cells, and bone-marrow-derived mesenchymal stem cells [61, 62]. Upon establishing this cell-CaP contact through integrins, a rearrangement of intracellular actin can occur, enabling signaling cascades involving mitogen-activated protein kinase (MAPK) signaling [63]. MAPK signaling is involved through Jagged/Notch gene activity in the release of common angiogenic growth factors in HUVECs, as well as the expression of their associated receptors [64]. Furthermore, the growth factors in the surrounding hemoderivative contribute strongly to tube formation as seen for the addition of the sole hemoderivatives.

Conversely to our work, a previous study has demonstrated pro-angiogenic effects after rinsing PL-preincubated CaP scaffolds, resulting in the secretion of pro-angiogenic growth factors by mesenchymal stromal cells [65]. However, the CaP materials used in this study were washed once in HBSS. Therefore, it is likely that different coating and washing procedures may influence the binding properties of platelet-derived proteins to CaP materials which have to be optimized in future studies. Thus, a comprehensive analysis of protein adsorption on hemoderivative-preincubated CaP materials in combination with their pro-angiogenic potential is crucial for biomaterial surface engineering using PLs as hemoderivative source.

## Conclusion

We state that all three hemoderivatives, as generated for this work, induced an angiogenic stimulus to HUVECs, with wPL showing the greatest effect. However, after adsorption on the CaP surfaces, the most abundant proteins identified by LC–MS were non-angiogenic or anti-angiogenic. In fact, we observed a reduction of pro-angiogenic VEGF, FGF-2 and PDGF-AB as measured by ELISA. The adsorbed proteins on the CaPs alone did not induce tube formation in HUVECs. Instead, tube formation was induced by keeping the hemoderivatives in the experiment. Consequently, biomaterial surfaces must be engineered to tightly adsorb pro-angiogenic growth factors and prevent dissipation out of wound cavities after implantation. We conclude that it is essential to analyze the protein adsorption on surfaces of synthetic bone substitute materials regarding pro-angiogenic responses. This correlation between material surface and the complex composition of adsorbed proteins must be considered to modify bone substitute materials with efficient regenerative capabilities. Overcoming the lack of angiogenesis or regenerative capabilities will lead to a reliable improvement of bone substitute materials to treat larger bone defects with higher success. Until then, the pro-angiogenic PL or wPL should not be washed away during the preparation of bone substitutes with these complex mixtures of proteins.

## Supplementary data

Supplementary data are available at *REGBIO* online.

## Supplementary Material

rbac044_Supplementary_Data
