# Supplementary material for: Proteomics reveals differential adsorption of angiogenic platelet lysate proteins on calcium phosphate bone substitute materials
Source: Regen Biomater. 2022 Jul 5;9:rbac044. doi: 10.1093/rb/rbac044 (PMC9348553; doi:10.1093/rb/rbac044)
Supplement: rbac044_Supplementary_Data [file rbac044_supplementary_data.zip › ProteomicsCaPs_P1_SI_ver6_20220527_Revision1.docx]

***Supporting information***

**Proteomics reveals differential adsorption of angiogenic platelet lysate proteins on calcium phosphate bone substitute materials**

**Richard da Costa Marques (R. M.)** **^a,b,1^, Johanna Simon (J. S.)** **^a,b,1^, Cyril d’Arros (C. D.) ^c,d^, Katharina Landfester (K. L.)** **^b^, Kerstin Jurk (K. J.) ^e,2^, Volker Mailänder (V.M.)  ^a,b,2*^**

^a^ Dermatology Clinic, University Medical Center of the Johannes Gutenberg-University Mainz, Langenbeckstr. 1, 55131 Mainz, Germany.

^b^ Max Planck Institute for Polymer Research, Ackermannweg 10, 55128 Mainz, Germany.

^c^ INSERM, UMR 1229, Regenerative Medicine and Skeleton, ONIRIS, Université de Nantes, 44042 Nantes, France

^d^ Biomatlante - Advanced Medical Solutions Group Plc, 44360 Vigneux-de-Bretagne, France.

^e^ Center for Thrombosis and Hemostasis (CTH), University Medical Center of the Johannes Gutenberg-University Mainz, Langenbeckstrasse 1, 55131, Mainz, Germany.

^1^ shared first authorship

^2^  shared last authorship

*Corresponding author

(E-Mail: mailaend@mpip-mainz.mpg.de, phone: +49 6131 378-248)

**Supporting Figures**


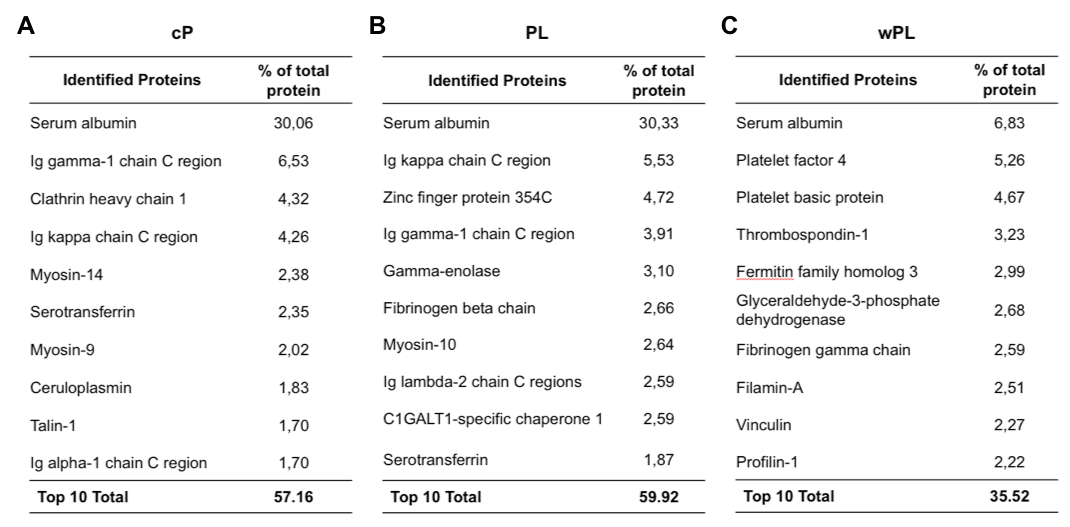


**Figure S1: The ten most abundant proteins identified by LC-MS in the hemoderivative protein sources.** (A-C) The 10 most abundant proteins in citrate Plasma (cP), platelet lysate in plasma (PL) and lysate of washed platelets (wPL) are listed. The values represent the percentage of total proteins identified.


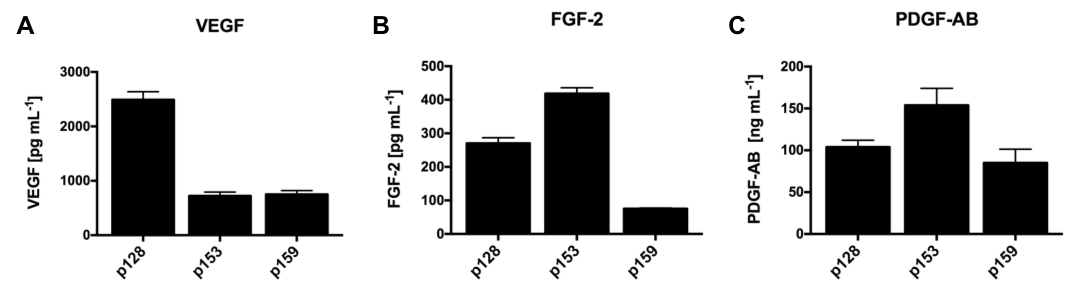


**Figure S2: Concentration of pro-angiogenic growth factors is donor-dependent in human washed platelet lysates.** Washed platelet lysates from three different donors were evaluated for angiogenesis-involved growth factors. The concentration of VEGF, FGF‑2, and PDGF‑AB was measured by ELISA (data is shown as mean ± SD, n=4).


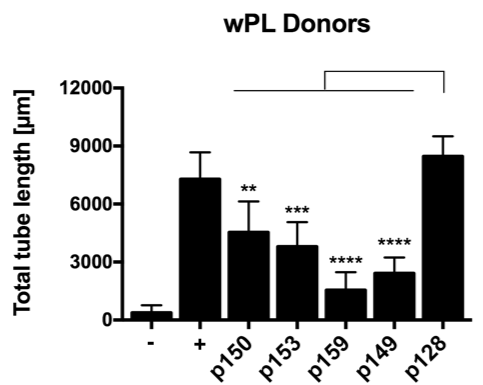


**Figure S3: Pro-angiogenic response in tube formation assays is donor-dependent for human washed platelet lysates.** The proangiogenic effect of washed platelet lysates from five different donors was analyzed by tube formation assays with HUVECs. HUVECs were incubated with the same concentration of wPL from five different donors on Geltrex^TM^ LDEV-Free Reduced Growth Factor Basement Membrane Matrix for 18 h. Total tube length was evaluated with ImageJ and the plugin Angiogenesis Analyzer (- = negative control: medium without LVES, + = positive control: medium with LVES; data is shown as mean ± SD, n= 3-9).


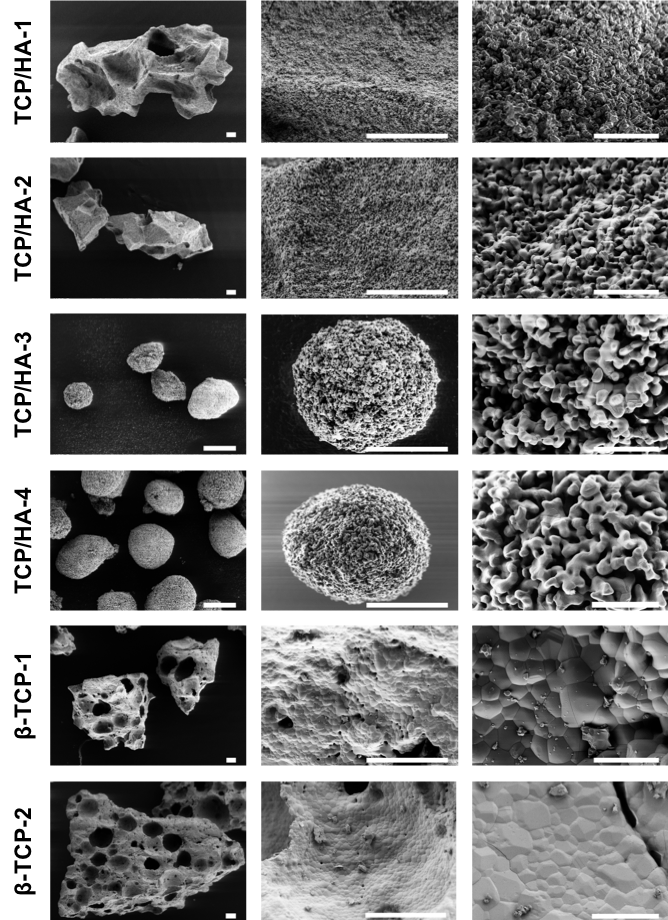


**Figure S4: SEM images of CaP materials.** Surface characterization in three magnifications. The left row scale bars represent 100 µm, the middle row scale bars represent 50 µm, and the right row scale bars represent 10 µm.


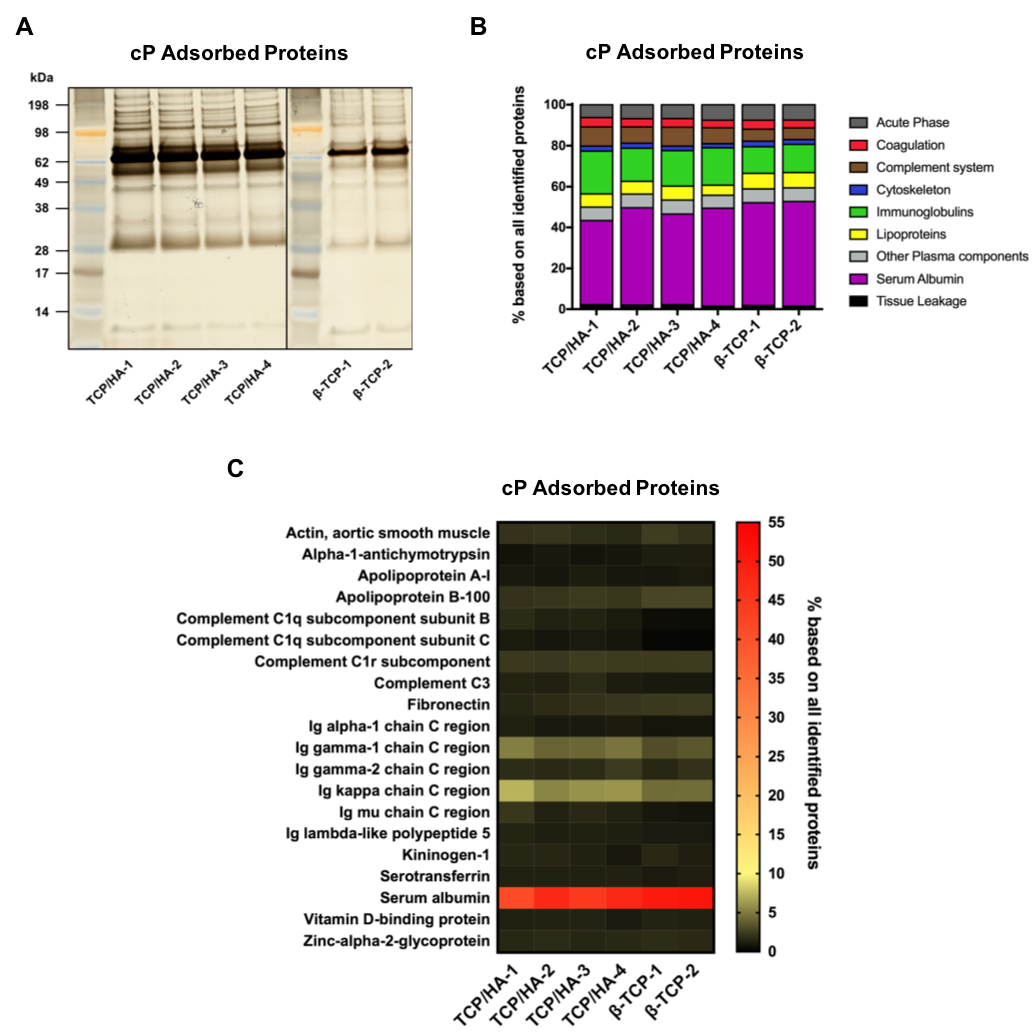


**Figure S5: Analysis of adsorbed proteins from human plasma on CaP surfaces.** CaP materials were incubated for 1 h in cP. After three subsequent washing steps, proteins were desorbed with 2% SDS. (A) SDS-PAGE and silver staining were performed for adsorbed proteins on different CaP surfaces incubated in cP. (B) Adsorbed proteins on CaP surfaces from cP were analyzed by quantitative LC-MS proteomics and identified proteins were classified into nine different protein groups. The bars indicate the percentage based on all identified proteins. (C) The heat map show the 20 most abundant proteins for TCP/HA 4 compared to the other CaP surfaces. The values represent the percentage based on all identified proteins.
