# Supplementary material for: Proteomics reveals differential adsorption of angiogenic platelet lysate proteins on calcium phosphate bone substitute materials
Source: Regen Biomater. 2022 Jul 5;9:rbac044. doi: 10.1093/rb/rbac044 (PMC9348553; doi:10.1093/rb/rbac044)
Supplement: rbac044_Supplementary_Data [file rbac044_supplementary_data.zip › ProteomicsCaPs_P1_ResponsetoRev_20220527_Revision1_VM.docx]

**Response to the reviewers**

**Reviewer #1**

**Comments to the Author:**

Protein adsorption on calcium phosphates (CaP), which evokes biological responses and shapes the interactions of biomaterials with the surrounding biological environment. Such topic is interesting and important. However, the manuscript should be revised to address the following issues:

**Question 1.1:** Fig 4c, Protein adsorption on CaPs should be summarized into a table for a more intuitive comparison and comparison of the adsorption changes of the three proteins on the surface of the six materials.

**Answer 1.1:**

We thank the reviewer for the suggestion. To provide a better overview of the quantified protein amounts that adsorbed on the CaP surfaces, we created a table with the values. The following table is now incorporated in the manuscript as Figure 4D. Additionally, we changed the axis description of Figure 4C to “µg protein per mg CaP” to fit Figure 4D. Text changes appear in yellow highlighting.

(D) Quantification of adsorbed proteins on CaPs, shown as a table, as seen in (C) (data is shown as mean ± SD, n=2-7).

**Question 1.2:** CP, PL wPL exhibited different absorption behaviors on 6 materials. If SSA is not the key factor, what are the key factors affecting the different adsorption capacity of these proteins on the material surface? It is important to describe the results, but the analysis and discussion of the mechanism behind the results is more important.

**Answer 1.2:**

We thank the reviewer for this comment to explain the driving key factors that shape the different protein adsorption outcomes.

We observed and reported that, overall, the materials with a higher SSA (and smaller crystal size) adsorb a higher amount of protein when incubated in PL or wPL. This trend was visible for wPL only in the case of the TCP/HA samples. The influence of SSA was stated and referenced in the manuscript (Duan et. al., 2017; Yuan et. al., 2010). The higher adsorption of wPL proteins in the case of β-TCP1 in comparison to the TCP/HA samples can be explained by the different chemical composition and the higher microporosity, which were also reported to be influential in protein adsorption processes (Wang et. al., 2012; Lee et. al., 2014).

For cP, we report a comparable amount of adsorbed protein between all CaPs with no trend connected to the CaP’s distinct physicochemical properties. Similar results have been observed previously (Wang et. al., 2014). It is accepted that the protein composition can overall affect adsorption processes (Wang et. al., 2012). Here, we must point out that the compositional nature between cP and the two other sources, PL and wPL, vary greatly. Both PL and wPL are lysates from platelets and contain a great variety of membrane proteins, lipids, and metabolites that adsorb on the surfaces of CaPs. Plasma is comprised of a majority of secreted and globular proteins with a far lower abundance of integral cellular parts. This key difference can indeed allow a distinct protein adsorption profile in the case of cP.

We refined the manuscript at the respective positions as follows:

**Results**

Interestingly, the highest concentration of adsorbed proteins was consistently measured for all materials after being incubated in wPL when compared to the other sources (Fig. 4C/D). For cP, we observed similar amounts of protein regardless of the used CaP surface. On the other hand, we noticed a decreasing trend of protein amount in the case of PL, which was following the decreasing SSA for the materials, reversibly the increasing crystal size. This same trend was observed for wPL in the case of the TCP/HA samples.

**Discussion**

Using the same workflow, including mass spectrometry-based proteomic analysis, we investigated the complex protein composition of the hemoderivatives adsorbed on different CaP materials, which revealed significant differences. Despite differences in the physicochemical properties of the CaP surfaces, we quantified comparable amounts of protein per amount of CaP in the case of cP and wPL but observed a decrease in protein amount with a decrease of the material’s SSA for adsorbed PL and wPL. For the latter protein source, the trend was observed for the TCP/HA samples. The impact of the SSA on protein adsorption was described in the literature [1, 2]. In any case, the protein adsorption on biomaterials represents a highly complex process that is driven by multiple factors and, therefore, challenging to predict. As described through experimental observations, adsorption on CaPs is influenced on one hand by the material’s physicochemical properties such as the surface area, the chemical composition, hydrophobicity, and the topography. On the other hand, the proteins’ properties, such as size, charge, and structure add a secondary dimension of influence [3, 4]. Complex protein mixtures as the herein described hemoderivative protein sources can therefore bring a variation to the adsorption result. Despite having a lower SSA, β-TCP-1 adsorbed more protein from wPL than the TCP/HA samples with a higher SSA. Seemingly, the higher microporosity and the chemical composition influence this result. For the cP-related adsorption, it is necessary to point out that plasma proteins are vastly different from lysate-derived proteins. While both lysates contain a significant amount of cellular components, such as membrane proteins, lipids, and metabolites, plasma is comprised mainly of secreted and globular proteins. This difference in composition results in similar protein amounts adsorbed throughout all CaP samples despite their physicochemical features. Similar observations were made in a study when incubating various CaP in serum [5]. Accordingly, the biomaterials physicochemical properties must be considered together with the composition of the protein source to evaluate the performance of protein adsorption.

**References**

1.	Duan R, Barbieri D, Luo X, Weng J, Bao C, de Bruijn JD, Yuan H. Variation of the bone forming ability with the physicochemical properties of calcium phosphate bone substitutes. *Biomater Sci* 2017;6:136-45.

2.	Yuan H, Fernandes H, Habibovic P, de Boer J, Barradas AM, de Ruiter A, Walsh WR, van Blitterswijk CA, de Bruijn JD. Osteoinductive ceramics as a synthetic alternative to autologous bone grafting. *Proc Natl Acad Sci U S A* 2010;107:13614-9.

3.	Wang K, Zhou C, Hong Y, Zhang X. A review of protein adsorption on bioceramics. *Interface focus* 2012;2:259-77.

4.	Lee W-H, Loo C-Y, Rohanizadeh R. A review of chemical surface modification of bioceramics: effects on protein adsorption and cellular response. *Colloids and Surfaces B: Biointerfaces* 2014;122:823-34.

5.	Wang J, Chen Y, Zhu X, Yuan T, Tan Y, Fan Y, Zhang X. Effect of phase composition on protein adsorption and osteoinduction of porous calcium phosphate ceramics in mice. *Journal of biomedical materials research Part A* 2014;102:4234-43.

**Question 1.3:** The Abstract and the Conclusion should be revised. Important results and data should be reflected in the abstract and conclusion.

**Answer 1.3:**

We thank the reviewer for the comment. The abstract and the conclusion were changed as follows (text changes appear in yellow highlighting):

**Abstract**

Protein adsorption on biomaterials for bone substitution, such as calcium phosphates (CaP), evokes biological responses and shapes the interactions of biomaterials with the surrounding biological environment. Proteins adsorb when CaP materials are combined with growth factor rich hemoderivatives prior to implantation to achieve enhanced angiogenesis and stimulate new bone formation. However, the identification of the adsorbed proteins and their angiogenic effect on bone homeostasis remain incompletely investigated. In this study, we analyzed the adsorbed complex protein composition on CaP surfaces when using the hemoderivatives plasma, platelet lysate in plasma (PL), and washed platelet lysate proteins (wPL). We detected highly abundant, non-regenerative proteins and anti-angiogenic proteins adsorbed on CaP surfaces after incubation with PL and wPL by liquid chromatography and mass spectrometry (LC-MS) proteomics. Additionally, we measured a decreased amount of adsorbed pro-angiogenic growth factors. Tube formation assays with human umbilical endothelial cells (HUVECs) demonstrated that the CaP surfaces only stimulate an angiogenic response when kept in the hemoderivative medium but not after washing with PBS. Our results highlight the necessity to correlate biomaterial surfaces with complex adsorbed protein compositions to tailor the biomaterial surface towards an enrichment of pro-angiogenic factors.

**Conclusion**

We state that all three hemoderivatives, as generated for this work, induced an angiogenic stimulus to HUVECs, with wPL showing the greatest effect. However, after adsorption on the CaP surfaces, the most abundant proteins identified by LC-MS were non-angiogenic or anti-angiogenic. In fact, we observed a reduction of pro-angiogenic VEGF, FGF-2, and PDGF-AB as measured by ELISA. The adsorbed proteins on the CaPs alone did not induce tube formation in HUVECs. Instead, tube formation was induced by keeping the hemoderivatives in the experiment. Consequently, biomaterial surfaces must be engineered to tightly adsorb pro-angiogenic growth factors and prevent dissipation out of wound cavities after implantation. We conclude that it is essential to analyze the protein adsorption on surfaces of synthetic bone substitute materials regarding pro-angiogenic responses. This correlation between material surface and the complex composition of adsorbed proteins must be considered to modify bone substitute materials with efficient regenerative capabilities. Overcoming the lack of angiogenesis or regenerative capabilities will lead to a reliable improvement of bone substitute materials to treat larger bone defects with higher success. Until then, the pro-angiogenic PL or wPL should not be washed away during the preparation of bone substitutes with these complex mixtures of proteins.

**Question 1.4:** References should be updated to recent publications.

Regenerative Biomaterials, 2018, 5(1): 35-41;

Regenerative Biomaterials, 2021, 8(3), rbab022;

Regenerative Biomaterials, Volume 6, Issue 1, February 2019, Pages 13–19;

Regenerative Biomaterials, Volume 6, Issue 4, August 2019, Pages 241–247.

These related literature should be cited.

**Answer 1.4:**

We thank the reviewer for the suggestion. We updated the introduction and included the four recommended works as follows (text changes appear in yellow highlighting):

**Introduction**

Differences in adsorption characteristics of proteins on biomaterial surfaces must be considered for the engineering of biomaterials to enhance their regenerative potential [1-3]. For bone substitution, biomaterial engineering has put forth a variety of synthetic bone substitute materials [4, 5]. Among these, calcium phosphates (CaP) and other synthetic bone substitute materials are more frequently used for smaller bone defects [5-7]. CaPs represent an absorbable and bioactive biomaterial platform with versatile and promising functionalization approaches to improve regenerative effects after application [8-11]. However, the application of CaPs in larger bone defects has been limited because of their inherent lack of growth factors [6, 12]. This lack of growth factors results in the inefficiency of CaPs to stimulate the formation of new bone [12]. To improve the formation of new bones, a crucial process is an efficient angiogenesis, the sprouting of new blood vessels [12, 13].

**References**

1.	Horbett TA. Protein Adsorption on Biomaterials. In: Biomaterials, interfacial phenomena and applications. Washington, D.C.: *American Chemical Society*, 1982, 233-44.

2.	Wang K, Zhou C, Hong Y, Zhang X. A review of protein adsorption on bioceramics. *Interface Focus* 2012;2:259-77.

3.	Horbett TA. The role of adsorbed proteins in tissue response to biomaterials. In: Ratner BD (ed). Biomaterials science : an introduction to materials in medicine. Amsterdam ; Boston: *Elsevier Academic Press*, 2004, 237-2346.

4.	Bauer TW, Muschler GF. Bone graft materials. An overview of the basic science. *Clin Orthop Relat Res* 2000:10-27.

5.	Kurien T, Pearson RG, Scammell BE. Bone graft substitutes currently available in orthopaedic practice: the evidence for their use. *Bone Joint J* 2013;95-B:583-97.

6.	Wang WY, K.W.K. Bone grafts and biomaterials substitutes for bone defect repair: A review. *Bioactive Materials* 2017;2:224 - 47.

7.	Fernandez de Grado G, Keller L, Idoux-Gillet Y, Wagner Q, Musset AM, Benkirane-Jessel N, Bornert F, Offner D. Bone substitutes: a review of their characteristics, clinical use, and perspectives for large bone defects management. *J Tissue Eng* 2018;9:2041731418776819.

8.	Zhou K, Ren X, Zhao M, Mei X, Zhang P, Chen Z, Zhu X. Promoting proliferation and differentiation of BMSCs by green tea polyphenols functionalized porous calcium phosphate. *Regenerative Biomaterials* 2017;5:35-41.

9.	Wang D, Zhang P, Mei X, Chen Z. Repair calvarial defect of osteoporotic rats by berberine functionalized porous calcium phosphate scaffold. *Regenerative Biomaterials* 2021;8.

10.	Meng D, Dong L, Yuan Y, Jiang Q. In vitro and in vivo analysis of the biocompatibility of two novel and injectable calcium phosphate cements. *Regenerative Biomaterials* 2018;6:13-19.

11.	Kjalarsdóttir L, Dýrfjörd A, Dagbjartsson A, Laxdal EH, Örlygsson G, Gíslason J, Einarsson JM, Ng C-H, Jónsson H, Jr. Bone remodeling effect of a chitosan and calcium phosphate-based composite. *Regenerative Biomaterials* 2019;6:241-47.

12.	Malhotra A, Habibovic P. Calcium Phosphates and Angiogenesis: Implications and Advances for Bone Regeneration. *Trends Biotechnol* 2016;34:983-92.

13.	Hankenson KD, Dishowitz M, Gray C, Schenker M. Angiogenesis in bone regeneration. *Injury* 2011;42:556-61.

**Reviewer #2**

**Comments to the Author:**

This paper reports on analysis of highly abundant, non-regenerative proteins and anti-angiogenic proteins which were adsorbed on CaP surfaces after incubation with PL and wPL, respectively using LC-MS proteomics approach. There are several important points missing in the manuscript.

**Question 2.1:** Authors mentioned that "Interestingly, the protein composition was similar across the different materials when incubated in the same hemoderivative source" in page 15. Please explain this result. Actually, we only obtain molecular weight information from SDS-PAGE.

**Answer 2.1:**

We thank the reviewer for the comment. Indeed, Figures 5A/B and Figure S5A show SDS-PAGEs and consequently reveal exclusively information on the protein composition by the molecular weight. Therefore, we changed the text at this part and described only the observations of the similar band patterns on the SDS-PAGE across the materials. However, the similar protein composition among the materials (when incubated in the same protein source) is finally only confirmed by proteomics. We can observe this for cP in Figure 5B/C and PL and wPL in Figure 5C/D and Figure 6. We now emphasized this in the manuscript. We changed the manuscript and marked the changes with yellow highlights as follows:

**Results**

The band pattern of the adsorbed proteins, as seen on SDS-PAGE, differed slightly from their original hemoderivative protein sources’ pattern. The protein pattern complexity was slightly reduced for wPL-related protein adsorption compared to cP and PL, respectively (Fig. 5A/B, Fig. S5A). Differences were more pronounced when the adsorbed proteins from wPL were compared to the absorbed proteins from cP and PL. Both cP and PL showed their strongest band at ~66 kDa, suggesting the rough molecular weight of serum albumin [1]. The strongest band for wPL was defined at ~42 kDa, suggesting β-actin [1, 2]. Interestingly, the band pattern was similar across the different materials when incubated in the same hemoderivative source. Only slight differences were observed on the gel between TCP/HA materials and β-TCP.

We employed LC-MS measurements to further characterize the complex adsorbed protein composition. Using the same protein functional classes as shown for the hemoderivatives, large differences in adsorbed proteins were revealed depending on the utilized hemoderivative. After the incubation in cP, the most abundant protein was serum albumin, followed by immunoglobulins (Fig. S5B). Likewise, serum albumin remained the highest protein in PL, although decreased. The other protein functional classes in PL, such as coagulation, complement, cytoskeleton, and immunoglobulins were distributed more evenly (Fig. 5C). The protein functional class distribution of wPL was quite distinct compared to cP and PL. Here, cytoskeleton proteins were found to be the most abundant group with more than 60% on every CaP surface used, followed by tissue-leakage proteins and coagulation (Fig. 5D). Generally, the protein composition by protein functional classes did not differ much between the different CaP materials within the same adsorbed protein source.

**References**

1.	Anderson L, Anderson NG. High resolution two-dimensional electrophoresis of human plasma proteins. *Proc Natl Acad Sci U S A* 1977;74:5421-5.

2.	Coppinger JA, Cagney G, Toomey S, Kislinger T, Belton O, McRedmond JP, Cahill DJ, Emili A, Fitzgerald DJ, Maguire PB. Characterization of the proteins released from activated platelets leads to localization of novel platelet proteins in human atherosclerotic lesions. *Blood* 2004;103:2096-104.

**Question 2.2:** Why could the CaP surfaces stimulate an angiogenic response in the hemoderivative medium? Please provide the possible mechanism, such as MAPK signal pathway, et al. not simple different adsorbed proteins.

**Answer 2.2:**

We gratefully acknowledge the reviewer’s question about the underlying mechanism of tube formation by the CaP surfaces in our experiment. We observed a pro-angiogenic stimulus of the CaP materials within the hemoderivative medium (Figure 8) but not by the materials alone or simply by the adsorbed proteins only (Figure 7B). For this reason, we want to highlight the combination of both components within the experiment as seen in Figure 8. While the addition of growth-factor rich hemoderivates did induce tube formation, we could observe the inclusion of the CaPs in the tubular structures of the HUVECs, indicating an interaction between cells and CaPs. Accordingly, we extended the discussion to review this aspect more thoroughly. The changes are highlighted in yellow and were implemented as follows:

**Discussion**

The observed lacking pro-angiogenic response of HUVECs after incubation with CaP materials adsorbed with proteins from platelet lysates might be due to a decreased binding affinity of the growth factors VEGF, FGF-2, PDGF-AB and increased binding affinity of the anti-angiogenic protein platelet factor 4 for the CaP materials, which were washed with PBS. Potentially, this washing procedure in the absence of divalent cations, e.g. Ca2+ and Mg2+ ions, may affect the types and amount of adsorbed proteins as non-washed CaP surfaces kept in hemoderivative protein source, still induced angiogenesis of cultured HUVECs. However, all three tested plasma and platelet protein sources showed similar pro-angiogenic effects compared to the positive control (LVES-supplemented medium). Additionally, we observed an inclusion of the CaPs in the HUVEC’s tubular structures, demonstrating an interaction between the cells and the CaPs. This adhesion of HUVECs on CaPs was already described in the literature [1]. It can be assumed that integrins on HUVECs establish contact with several adsorbed proteins with integrin-binding domains on the CaPs, such as fibronectin or vitronectin. This formation of a focal contact between CaPs and cells through protein contacts was experimentally explained for marrow stromal cells, osteosarcoma cells, and bone-marrow-derived mesenchymal stem cells [2, 3]. Upon establishing this cell-CaP contact through integrins, a rearrangement of intracellular actin can occur, enabling signaling cascades involving mitogen-activated protein kinase (MAPK) signaling [4]. MAPK signaling is involved through Jagged/Notch gene activity in the release of common angiogenic growth factors in HUVECs, as well as the expression of their associated receptors [5]. Furthermore, the growth factors in the surrounding hemoderivative contribute strongly to tube formation as seen for the addition of the sole hemoderivatives.

Conversely to our work, a previous study has demonstrated pro angiogenic effects after rinsing PL preincubated CaP scaffolds, resulting in the secretion of pro angiogenic growth factors by mesenchymal stromal cells [6]. However, the CaP materials used in this study were washed once in HBSS. Therefore, it is likely that different coating and washing procedures may influence the binding properties of platelet-derived proteins to CaP materials which have to be optimized in future studies. Thus, a comprehensive analysis of protein adsorption on hemoderivative preincubated CaP materials in combination with their pro-angiogenic potential is crucial for biomaterial surface engineering using platelet lysates as hemoderivate source.

**References**

1.	Chen Y, Wang J, Zhu X, Tang Z, Yang X, Tan Y, Fan Y, Zhang X. Enhanced effect of β-tricalcium phosphate phase on neovascularization of porous calcium phosphate ceramics: in vitro and in vivo evidence. *Acta biomaterialia* 2015;11:435-48.

2.	Kilpadi KL, Sawyer AA, Prince CW, Chang PL, Bellis SL. Primary human marrow stromal cells and Saos‐2 osteosarcoma cells use different mechanisms to adhere to hydroxylapatite. *Journal of Biomedical Materials Research Part A: An Official Journal of The Society for Biomaterials, The Japanese Society for Biomaterials, and The Australian Society for Biomaterials and the Korean Society for Biomaterials* 2004;68:273-85.

3.	Sawyer A, Hennessy K, Bellis S. Regulation of mesenchymal stem cell attachment and spreading on hydroxyapatite by RGD peptides and adsorbed serum proteins. *Biomaterials* 2005;26:1467-75.

4.	Chen X, Wang J, Chen Y, Cai H, Yang X, Zhu X, Fan Y, Zhang X. Roles of calcium phosphate-mediated integrin expression and MAPK signaling pathways in the osteoblastic differentiation of mesenchymal stem cells. *Journal of Materials Chemistry B* 2016;4:2280-89.

5.	Kiec-Wilk B, Grzybowska-Galuszka J, Polus A, Pryjma J, Knapp A, Kristiansen K. The MAPK-dependent regulation of the Jagged/Notch gene expression by VEGF, bFGF or PPARγ mediated angiogenesis in HUVEC. *Journal of Physiology and Pharmacology* 2010;61:217.

6.	Leotot J, Coquelin L, Bodivit G, Bierling P, Hernigou P, Rouard H, Chevallier N. Platelet lysate coating on scaffolds directly and indirectly enhances cell migration, improving bone and blood vessel formation. *Acta Biomater* 2013;9:6630-40.
